# Association between Charlson comorbidity index and complications of endoscopic resection of gastric neoplasms in elderly patients

**DOI:** 10.1186/s12876-020-01360-6

**Published:** 2020-07-09

**Authors:** Sunmin Kim, Dong Hyun Kim, Seon-Young Park, Chang Hwan Park, Hyun Soo Kim, Sung Kyu Choi, Jong Sun Rew

**Affiliations:** Division of Gastroenterology and Hepatology, Department of Internal Medicine, Chonnam National Medical School, Gwangju, South Korea

**Keywords:** Endoscopic mucosal resection, Aged, Comorbidity, Stomach neoplasms

## Abstract

**Background:**

Although endoscopic resection is safe and effective for gastric epithelial neoplasms, information is limited on its efficacy and safety in extremely elderly patients who have various comorbidities. Further, the relationship between comorbidities and complications of endoscopic resection is not well established. Therefore, we aimed to evaluate the efficacy and safety of endoscopic resection of gastric epithelial neoplasms in extremely elderly patients.

**Methods:**

From October 2008 to December 2017, 4475 consecutive patients underwent endoscopic resection of gastric epithelial neoplasms. Among them, 242 were 75 years or older. We assessed Charlson comorbidity index (CCI) scores, procedural outcomes, and procedure- and sedation-related complications related to endoscopic resection.

**Results:**

Mean patient age was 78.7 ± 3.2 years. Of the 242 patients, 124 (51.2%) had low-grade dysplasia and 112 (46.3%) had adenocarcinoma. The most common comorbidity was hypertension (55.4%), followed by diabetes (23.1%). The mean CCI score was 1.67 ± 1.43. Sixty patients (24.8%) had a CCI score ≥ 3. During the procedure, 10 (4.1%) patients had desaturation that recovered by flumazenil use with mask (*n* = 2) or Ambu bag (*n* = 3) ventilation. During subsequent admission, atelectasis or pneumonia occurred in 45 (18.6%) patients, post-procedural bleeding in 12 (5.0%), and perforation in 3 (1.2%). Respiratory complications were more common in patients with a CCI score ≥ 3 (20/60, 33.3%) than in those with a CCI score < 3 (25/182, 13.7%, *P* = 0.002).

**Conclusions:**

CCI score is related to respiratory complications of endoscopic resection in extremely elderly patients. Endoscopic resection must be performed cautiously, particularly in elderly patients with a high CCI score, to prevent respiratory complications.

## Background

Endoscopic resection is safe and effective for the treatment of gastric epithelial neoplasms and has a low risk of procedure-related complications [1–3]. In increasingly aged societies, a growing number of endoscopic treatments are performed for the elderly, and several reports have shown contrasting treatment outcomes and complications of endoscopic resection in elderly and non-elderly patients [4–8].

Elderly patients, especially those aged 75 years or older (extremely elderly patients), have various comorbidities and functional disabilities that influence daily life [9, 10]. Although several studies have reported that endoscopic mucosal resection (EMR) and endoscopic submucosal dissection (ESD) are safe and reliable in the elderly [4–8], endoscopy itself carries risks in elderly patients, particularly those with renal or respiratory comorbidities [11, 12].

In the real-world clinical setting, endoscopists must consider comorbidities, performance status (PS), and expected survival in elderly patients. The relationship between comorbidities and complications of endoscopic resection in elderly patients is not well established. The Charlson comorbidity index (CCI) has been widely utilized to measure the burden of complex comorbidities by health researchers [13]. The CCI predicts mortality by classifying or weighting comorbidities and has been validated in various disease subgroups, including stroke, cardiac disease, renal disease, and liver disease [14–17]. However, no studies to date have examined the relationship between CCI score and incidence of complications after endoscopic resection of gastric epithelial neoplasms in elderly patients.

Here we aimed to evaluate the CCI and efficacy and safety of endoscopic resection of gastric epithelial neoplasms in extremely elderly patients.

## Methods

### Study population

This retrospective study was conducted in accordance with the Ethical Guidelines of the Declaration of Helsinki. This study was approved by the Institutional Review Board of Chonnam National University Hospital (no. CNUH-2018-236).

From October 2008 to December 2017, a total of 4475 consecutive patients underwent endoscopic resection of gastric epithelial neoplasms in our center. Among them, 242 aged 75 years or older were included in this study. The patients underwent ESD or EMR for gastric epithelial neoplasms. We reviewed their medical records and extracted information about their demographic and clinical characteristics, procedural outcomes, and procedure- or sedation-related complications.

### Assessment of CCI scores before endoscopic procedure

The components of CCI include: previous myocardial infarction, congestive cardiac failure, peripheral vascular disease, cerebrovascular accident, dementia, chronic obstructive pulmonary disease, connective tissue disease, peptic ulcer disease, diabetes, renal disease, hemiplegia, leukemia, lymphoma, solid tumor with or without metastatic disease, liver disease, and acquired immunodeficiency syndrome status. We calculated the CCI by summing the weights of all comorbid parameters. The total CCI score was 0–33. We divided the patients into those with a CCI < 3 and those with a CCI ≥ 3.

### Performance status

We reviewed the patients’ medical records to investigate their social history, body mass index, and comorbidities. Based on these data, the performance status of all enrolled patients was evaluated using the American Society of Anesthesiologists-Performance Status and Eastern Cooperative Oncology Group-Performance Status.

### Endoscopic procedure

Endoscopists determined the methods of removal for the lesions (EMR or ESD) according to shape, size, presence of fibrosis, or presence of lesion ulceration before endoscopic resection. For ESD or EMR, patients were placed in the left lateral decubitus position. Each endoscopic procedure was performed using a single-channel upper gastrointestinal endoscope with transparent hood under CO_2_ insufflation.

### Anticoagulants or antiplatelet agents

Anticoagulant and antiplatelet agent therapy was discontinued for endoscopic resection according to the recommended cessation period [18]. The endoscopic procedure was evaluated as a high risk procedure that could cause bleeding, and the discontinuation of the drug was determined individually according to the patient’s pre-existing thromboembolic condition.

### Anesthesia procedure

All procedures were performed with use of endoscopist-directed sedation. Patients were sedated with midazolam and pethidine with or without propofol. The target sedation level was mild to moderate [19]. For sedation induction, midazolam 3 mg and pethidine 25 mg were intravenously injected. Thereafter, additional propofol, midazolam, or pethidine was intravenously injected to ensure adequate sedation or pain control. Oxygen was supplied at a constant level of 2 L/min via a nasal prong during the procedure, and sedative medication doses were adjusted according to the vital signs of patients. We monitored blood pressure, heart rate, and oxygen saturation during the procedure.

### Outcome measures

The primary outcome was procedure- and sedation-related complications, while the secondary outcomes were procedure time and complete resection rate.

#### Procedure-related complications

Procedure-related bleeding was defined as bleeding requiring transfusion or emergency endoscopy or that reduced the hemoglobin level by more than 2 g/dL following the procedure. We defined immediate bleeding as bleeding occurring within 24 h after the endoscopic resection and delayed bleeding as gastrointestinal bleeding occurring later than 24 h after the endoscopic resection [20, 21]. Procedure-related perforation was defined as endoscopically observed extraluminal space or intra-abdominal free air on chest radiography taken after the procedure [21].

#### Sedation-related complications

Sedation-related complications were divided into immediate complications (hypotension, arrhythmia, hypoxia) during endoscopy and post-procedural complications (respiratory complications such as atelectasis and pneumonia).

Hypotension during the procedure was defined as systolic blood pressure below 90 mmHg. Oxygen desaturation was defined as oxygen arterial saturation < 90% for at least 10 s. Supplemental oxygen was given to maintain oxygen arterial saturation > 90%. Bradycardia was defined as any episode of heart rate < 40 beats per minute.

All patients underwent pre-procedure chest radiography at the time of admission. Atelectasis was diagnosed by comparison of post-procedure and pre-procedure chest radiographic findings, regardless of clinical symptoms. Radiographic findings of atelectasis include direct signs such as crowding of pulmonary vessels, crowed air bronchogram, and displacement of interlobar fissure as well as indirect signs such as pulmonary opacification and elevation of the ipsilateral diaphragm. Pneumonia was defined as newly developed pulmonary infiltration with clinical symptoms such as cough, sputum, and fever with chilling. In these cases, proper antibiotics were administered.

#### Procedure time

Procedure time was defined as the time from the start of intravenous administration of the sedative agent to the time of endoscope extubation.

#### Complete resection

Complete resection was defined as follows: 1) tumor removed in one piece (en bloc resection) and horizontal/vertical margin was histologically free from tumorous glands; or 2) tumor removed in multiple pieces (piecemeal resection) and follow-up endoscopy revealed no recurrence for at least 1 year.

#### Follow up endoscopy

Follow-up endoscopy was performed 3–6 months after endoscopic resection, and follow-up endoscopy was performed every year thereafter. We defined tumor recurrence as local recurred or metachronous lesions in the stomach after 1 year after endoscopic resection of the primary lesion.

### Statistical analysis

Statistical analysis was performed using SPSS version 23.0 (SPSS Inc., Chicago, IL, USA). Continuous data are shown as mean ± standard deviation or median (range), while categorical data are shown as absolute and relative frequencies. Continuous variables were analyzed using Student’s t-test. Categorical data were examined using Fisher’s exact test or the chi-squared test. On multivariate analysis, binary logistic regression models with forward conditioning were used to investigate CCI-associated complications. The data included in the regression analysis are presented as crude or adjusted odds ratios with 95% confidence intervals (CIs). Variables with *P* values < 0.05 on the univariate analysis were selected for inclusion in the multivariate analysis.

## Results

### Patient demographics

The mean patient age was 78.7 ± 3.2 years. Of the 242 patients, 124 (51.2%) had low-grade dysplasia and 112 (46.3%) had carcinoma in situ or adenocarcinoma (Table 1). The most common comorbidity was hypertension (55.4%), followed by diabetes (23.1%) (Table 2). A total of 141 (58.3%) patients underwent ESD. Seventy-one (31%) patients underwent endoscopic resection of multiple lesions in a single session. The mean procedure time was 34.9 ± 23.7 min, and the mean hospital stay was 5.0 ± 1.7 days. Midazolam was used in all patients and propofol was used in 215 (88.8%) patients.

**Table 1:** Endoscopic and histologic findings in 242 elderly patients

Variables	
Number of lesions, *n* (%)	
Single	166 (68.6)
Multiple	76 (31.4)
Location of lesion, *n* (%)	
Upper 1/3	28 (11.5)
Mid 1/3	78 (32.2)
Lower 1/3	136 (56.2)
Macroscopic appearance, *n* (%)	
Flat/depressed	67 (27.7)
Elevated	175 (72.3)
Histologic findings, *n* (%)	
Low-grade dysplasia	124 (51.2)
Carcinoma in situ or adenocarcinoma	112 (46.3)
Other	6 (2.5)
Method of endoscopic resection, *n* (%)	
EMR	100 (41.3)
ESD	142 (58.7)
Mean procedure time, min, mean ± SD	34.9 ± 23.7
Method of Sedation	
Midazolam + Propofol+ Pethidine	215 (88.8)
Midazolam + Pethidine	27 (11.2)
Dose of sedative agents, mean ± SD	
Midazolam, mg	3.43 ± 1.16
Propofol, mg	112.0 ± 88.0

*EMR* endoscopic mucosal resection, *ESD* endoscopic submucosal dissection, *SD* standard deviation

**Table 2:** Baseline characteristics and comorbidities of elderly patients who underwent endoscopic resection

Variables		
Age, yrs., mean ± SD		78.7 ± 3.2
Female sex, *n* (%)		101 (41.7)
Body mass index, kg/m^2^, mean ± SD		23.7 ± 3.3
Comorbidities, variable CCI, *n* (%)	Weighting	
Acute myocardial infarction	1	30 (12.4)
Congestive heart failure	1	11 (4.5)
Peripheral vascular disease	1	3 (1.2)
Cerebrovascular accident	1	11 (4.5)
Dementia	1	5 (2.1)
Chronic pulmonary disease	1	27 (11.2)
Connective tissue disease	1	0 (0)
Ulcer disease	1	0 (0)
Mild liver disease	1	13 (5.4)
Hepatitis		8 (3.3)
Diabetes	1	56 (23.1)
Diabetes with end organ damage	2	0 (0)
Hemiplegia	2	0 (0)
Moderate or severe renal disease	2	3 (1.2)
Any tumor	2	31 (12.8)
Leukemia	2	0 (0)
Lymphoma	2	0 (0)
Moderate to severe liver disease	3	5 (2.1)
Metastatic solid tumor	6	0 (0)
Acquired immunodeficiency syndrome	6	0 (0)
Charlson comorbidity index, mean ± SD		1.67 ± 1.43
ASA-PS, mean ± SD		2.01 ± 0.49
ECOG-PS, mean ± SD		1.55 ± 0.55
Medication, *n* (%)		
Aspirin		70 (28.9)
Clopidogrel		10 (4.1)
Antithrombotic agents		1 (0.4)

*SD* standard deviation, *ASA-PS* American Society of Anesthesiologists Performance Status, *CCI* Charlson comorbidity index, *ECOG-PS* Eastern Cooperative Oncology Group Performance Status

### Charlson comorbidity index

**Fig. 1:**
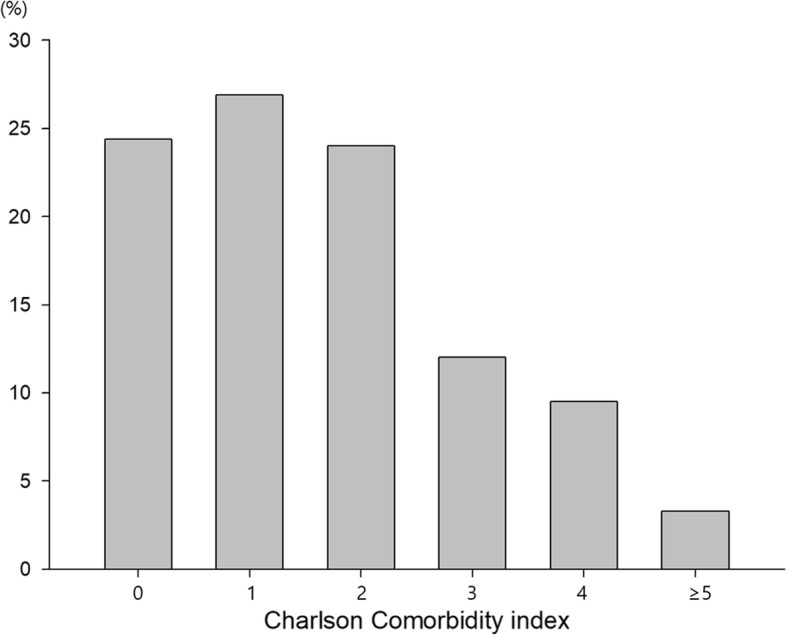
Percentage of patients by CCI score

The mean CCI score was 1.67 ± 1.43 (range, 0–7). Figure 1 shows the distribution of the CCI score in the 242 patients: 59 (24.4%) had a CCI score of 0, 65 (26.9%) had a CCI score of 1, 58 (24.0%) had a CCI score of 2, 29 (12.0%) had a CCI score of 3, 23 (9.5%) had a CCI score of 4, 6 (2.5%) had a CCI score of 5, 1 (0.4%) had a CCI score of 6, and 1 (0.4%) had a CCI score of 7. Thus, 182 patients had a low CCI score (< 3) and 60 had a high CCI score (≥3).

### Procedure-related complications

Procedure-related complications included immediate bleeding (4.1%), delayed bleeding (0.8%) and perforation (1.2%). There were no significant differences in procedure-related complications between the low and high CCI groups.

### Sedation-related complications

During the procedure, there were 10 (4.1%) patients with desaturation recovered by flumazenil with use mask (*n* = 2) or Ambu bag (*n* = 3) ventilation. No patient received endotracheal intubation during endoscopic procedure. There were no significant differences in the incidence of hypotension or hypoxia during the procedure between the low and high CCI groups.

**Fig. 2:**
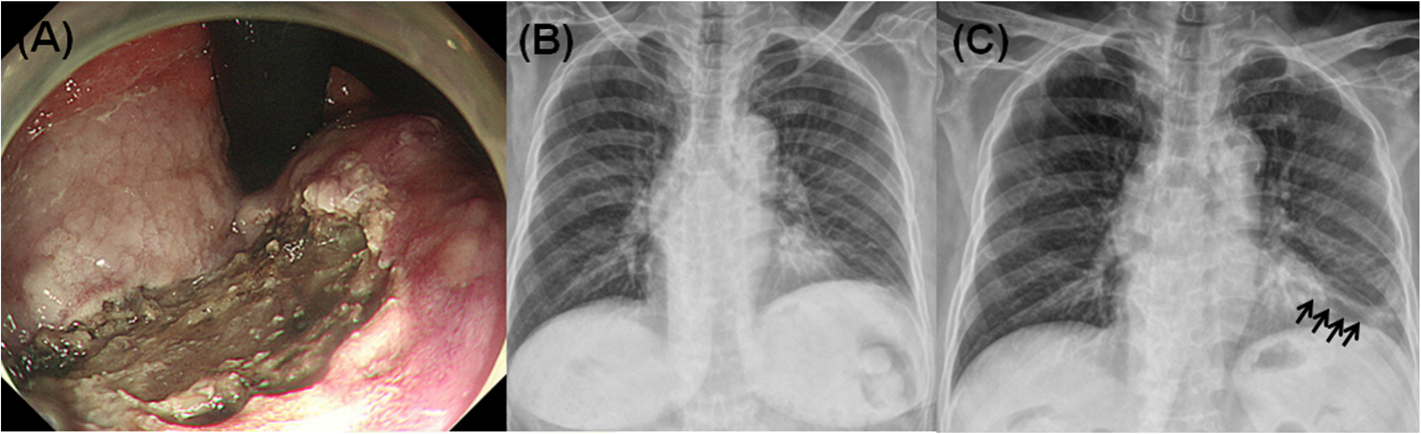
One example of atelectasis in an elderly patient (patient aged 75 years over who underwent endoscopic submucosal dissection). Photo of procedure **a**. X-ray before procedure **b** and 24 h after the procedure **c**

Post-procedural complications such as atelectasis or pneumonia occurred in 45 (18.6%) patients. In 4 patients, pneumonia was diagnosed due to fever, respiratory symptoms, and chest radiography abnormalities. These patients were treated with antibiotics and improved without sequelae. Most other patients improved spontaneously during the outpatient follow-up period (Fig. 2).

**Fig. 3:**
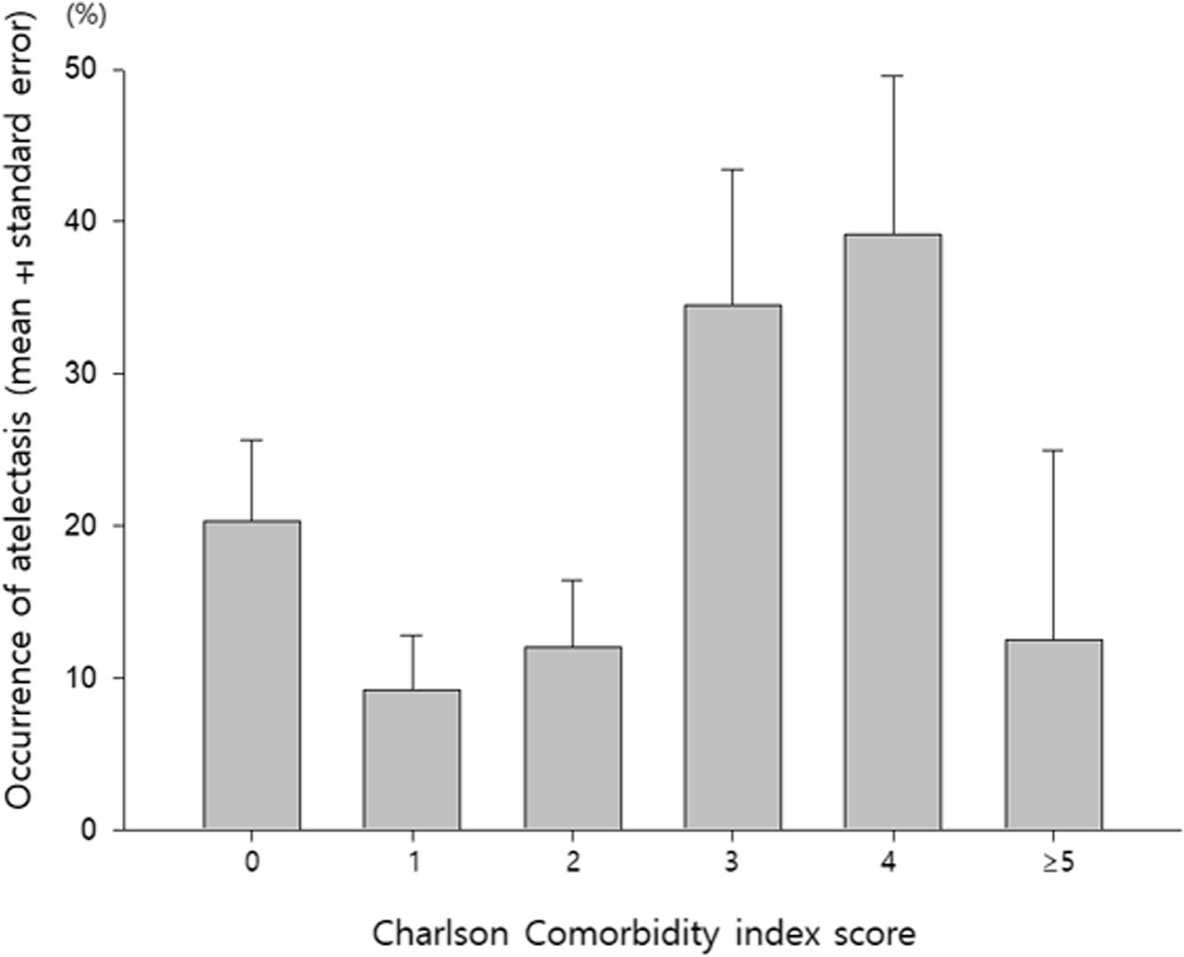
Atelectasis occurrence by CCI score (Data are expressed as mean ± standard error)

The incidence of respiratory complications such as atelectasis or pneumonia tended to increase with CCI score: 20.3% (*n* = 12/59) in patients with a score of 0, 9.2% (*n* = 6/65) in patients with a score of 1, 12.1% (*n* = 7/58) in patients with a score of 2, 34.5% (*n* = 10/29) in patients with a score of 3, 39.1% (*n* = 9/23) in patients with a score of 4, and 12.5% (*n* = 1/8) in patients with a score of 5 or more (Fig. 3). The incidence of atelectasis was 20/60 (33.3%) in patients with a CCI score ≥ 3 and 21/182 (11.5%) in patients with a CCI score < 3 (*P* < 0.01). Post-procedural pneumonia developed only in 4 patients of CCI < 3. The incidence of respiratory complications (atelectasis or pneumonia) was higher in the group with a CCI score ≥ 3 (Table 3).

**Table 3:** Treatment outcomes and complications by CCI score

	CCI < 3 (*n* = 182)	CCI ≥ 3 (*n* = 60)	*P* value
Age, mean ± SD	78.6 ± 3.1	79.0 ± 3.3	0.38
Sex (female)	81 (44.5)	20 (33.3)	0.13
Procedure time, min, median (range)	30 (4 ~ 150)	25 (5 ~ 120)	0.05
Resectability, *n* (%)			
En bloc resection	161 (88.5)	58 (96.7)	0.06
Piecemeal resection	21 (11.5)	2 (3.3)	
Method of sedation, *n* (%)			
Midazolam + Pethidine + Propofol	160 (87.9)	55 (91.7)	0.42
Midazolam + Pethidine	22 (12.1)	5 (8.3)	
Dose of sedative agents, mean ± SD			
Midazolam, mg	3.65 ± 0.95	3.39 ± 0.87	0.07
Propofol, mg	126.4 ± 79.2	132.6 ± 92.5	0.64
Procedure-related complications, *n* (%)			
Immediate bleeding	10 (5.5)	0	0.06
Delayed bleeding	2 (1.1)	0	0.42
Perforation	3 (1.6)	0	0.32
Sedation-related complications, *n* (%)			
Immediate complication			
Hypotension	1 (0.5)	0	0.57
Arrhythmia	37 (20.3)	14 (23.3)	0.62
Hypoxia	8 (4.4)	2 (3.3)	0.72
Hypopnea or apnea	1 (0.5)	0	0.57
Delayed complication			
Atelectasis	21 (11.5)	20 (33.3)	< 0.01
Pneumonia	4 (2.2)	0 (0)	0.25
Atelectasis or Pneumonia	25 (13.7)	20 (33.3)	< 0.01

*CCI* Charlson comorbidity index

Uni- and multivariate analyses showed that age and presence of a CCI score ≥ 3 were related to the incidence of atelectasis or pneumonia after endoscopic resection in extremely elderly patients. The adjusted odds ratio for patients with a CCI score ≥ 3 and the development of atelectasis or pneumonia was 3.103 (95% CI, 1.538–6.261, Table 4).

**Table 4:** Univariate and multivariate analysis for predicting atelectasis/pneumonia after endoscopic resection in elderly patients

Variables	cOR (95% CI)	*P* value	aOR (95% CI)	*P* value
Age, yrs	1.175 (1.065–1.296)	0.001	1.173 (1.061–1.297)	0.002
Sex (female)	0.781 (0.408–1.498)	0.458		
Body mass index, kg/m^2^	0.999 (0.904–1.105)	0.987		
CCI ≥ 3	3.140 (1.586–6.215)	0.001	3.103 (1.538–6.261)	0.002
Use of propofol	0.738 (0.242–2.251)	0.593		
Procedure time, min	1.012 (1.000–1.025)	0.056		

*cOR* crude odds ratio, *aOR* adjusted OR, *CCI* Charlson comorbidity index

#### Clinical outcomes

En bloc resection was achieved in 90.5% (219/242) of patients, while complete resection was achieved in 87.2% (211/242) of patients. Among 31 patients with incomplete resection, 12.9% (4/31) patients underwent additional operation and 3.2% (1/31) underwent additional endoscopic resection. There was no significant difference in the en bloc resection or complete resection rates between the patients with low and high CCI scores (Table 3). Tumor recurrence occurred in 3.8% (8/211) patients with complete resection. .

## Discussion

Our study demonstrated that: (1) endoscopic resection was effective and safe for extremely elderly (≥75 years of age) patients with gastric neoplasms under careful consideration; and (2) age and higher CCI score (≥3) were independent risk factors for atelectasis or pneumonia after endoscopic resection in extremely elderly patients.

Several studies have examined the treatment outcomes and safety of endoscopic resection in elderly patients, which showed no significant difference in treatment outcomes of endoscopic resection of EGC in elderly patients [4–6]. In our study including extremely elderly patients, the en bloc resection rate was 90.5% and the complete resection rate was 87.2%, similar to the rates reported in previous studies [4, 7, 20, 21]. Procedure-related bleeding and procedure-related perforation was observed in 4.9 and 0.4% of extreme elderly patients, respectively. These complication rates were comparable in those of other studies including elderly or non-elderly patients [7, 20]. Sedation-related complications such as intra-procedural hypotension and oxygen desaturation is known to be occurred more frequently in elderly patients [20, 22]. Furthermore, post-procedural respiratory complications such as atelectasis or pneumonia developed more frequently in elderly patients than in nonelderly patients [7, 20]. Tokioka et al. demonstrated that recovery time from respiratory complications was longer and performance score was higher after endoscopic resection of EGC in patients aged 65 years and older [4]. In our study, significant hypoxemia occurred in 4.1% and post-procedural respiratory complications occurred in 18.6% (atelectasis in 16.9% and pneumonia in 1.7%). Development of atelectasis in perioperative patients is associated with decreased lung compliance and increased pulmonary vascular resistance leading to lung injury and impairment of oxygenation [23]. Even though most patients with atelectasis recovered with conservative treatment in this study, persistent prolonged atelectasis after anesthesia is known to increase perioperative respiratory complications leading to significant consequence, especially in patients with underlying lung disease or cardiopulmonary dysfunction [23–26]. Therefore, early detection of atelectasis and effort for reversal of atelectasis in those patients may prevent significant periprocedural respiratory complication and improve clinical outcomes.

In recent studies, respiratory complication after endoscopic resection was found to be significantly related to the procedure time, the degree of sedation, and the amount of propofol use [27, 28]. In our study, respiratory complication tended to increase with increasing procedure time, but it was not statistically significant. In addition, there was no correlation between the use of propofol or the dose used, and an increase in respiratory complication.

Charlson et al. developed a scoring system to classify prognostic comorbidity and predict in-hospital and 1-year mortality rates [13]. Several studies reported that CCI predicted the prognosis of various diseases such as ischemic stroke [14], end-stage renal disease [15], cirrhosis [17], and lung cancer [29]. It could be adapted to patients in health care [30] and post-operative outcomes in acute cholecystitis [31]. In our study, CCI score was not related to procedure-related complications or immediate sedation-related complications such as hypoxemia or hypotension. However, post-procedural complications including atelectasis/pneumonia were frequently observed in extremely elderly patients with higher a CCI score (≥3). Therefore, we expect to apply CCI score to other advanced endoscopic procedures including endoscopic retrograde cholangio-pancreatography, endoscopic ultrasonography-guided intervention, submucosal tunneling endoscopic resection or enteroscopy in elderly patients to evaluate the risk for periprocedural complications.

This study has several limitations; first, this study might not have included patients with very severe or uncontrolled comorbidities due to its retrospective design. All enrolled patients could be selected since they were considered suitable candidates for endoscopic therapy by the endoscopist. However, it is not possible to design a prospective study that enrolls only patients with severe morbidities due to ethical concerns. Second, we could not get the information for effect of sedative agents, as clinician choose the sedative agents for the procedure according to risk assessment of procedural complication.

## Conclusions

In conclusion, although endoscopic resection is considered safe and effective for elderly patients, endoscopists must perform it cautiously, particularly in extremely elderly patients with a high CCI score, to prevent post-procedural respiratory complications such as pneumonia or atelectasis. In the future, we need to conduct a study to prevent postprocedural respiratory complication.

## Data Availability

The datasets used and/or analyzed during the current study available from the corresponding author on request.
